# Molecular Characterization and Survive Abilities of *Salmonella* Heidelberg Strains of Poultry Origin in Brazil

**DOI:** 10.3389/fmicb.2021.674147

**Published:** 2021-06-18

**Authors:** Roberta T. Melo, Newton N. Galvão, Micaela Guidotti-Takeuchi, Phelipe A. B. M. Peres, Belchiolina B. Fonseca, Rodrigo Profeta, Vasco A. C. Azevedo, Guilherme P. Monteiro, Bertram Brenig, Daise A. Rossi

**Affiliations:** ^1^Faculty of Veterinary Medicine, Federal University of Uberlândia, Uberlândia, Brazil; ^2^Ministry of Agriculture, Livestock and Supply, Rio de Janeiro, Brazil; ^3^Department of Genetics, Ecology and Evolution (GEE), Federal University of Minas Gerais, Belo Horizonte, Brazil; ^4^Institute of Veterinary Medicine, University of Göttingen, Göttingen, Germany

**Keywords:** biofilms, antimicrobial resistance, whole genome sequencing, virulence, salmonellosis

## Abstract

The aim of the study was to evaluate the genotypic and phenotypic characteristics of 20 strains of *S.* Heidelberg (*S*H) isolated from broilers produced in southern Brazil. The similarity and presence of genetic determinants linked to virulence, antimicrobial resistance, biofilm formation, and *in silico*-predicted metabolic interactions revealed this serovar as a threat to public health. The presence of the *ompC*, *invA*, *sodC*, *avrA*, *lpfA*, and *agfA* genes was detected in 100% of the strains and the *luxS* gene in 70% of them. None of the strains carries the *bla*_SHV_, *mcr-1, qnrA, qnrB*, and *qnrS* genes. All strains showed a multidrug-resistant profile to at least three non-β-lactam drugs, which include colistin, sulfamethoxazole, and tetracycline. Resistance to penicillin, ceftriaxone (90%), meropenem (25%), and cefoxitin (25%) were associated with the presence of *bla*_CTX–M_ and *bla*_CMY–2_ genes. Biofilm formation reached a mature stage at 25 and 37°C, especially with chicken juice (CJ) addition. The sodium hypochlorite 1% was the least efficient in controlling the sessile cells. Genomic analysis of two strains identified more than 100 virulence genes and the presence of resistance to 24 classes of antibiotics correlated to phenotypic tests. Protein-protein interaction (PPI) prediction shows two metabolic pathways correlation with biofilm formation. Virulence, resistance, and biofilm determinants must be constant monitoring in *S*H, due to the possibility of occurring infections extremely difficult to cure and due risk of the maintenance of the bacterium in production environments.

## Introduction

Salmonellosis is one of the main enteric bacterial diseases in humans. Its public health importance is mainly associated with food-borne infection, usually caused by the consumption of chicken meet that are often colonized by zoonotic serovars (Elhariri et al., 2017). In the United States, *Salmonella* spp. affects approximately 1.2 million people each year, resulting in 23,000 hospitalizations and 450 deaths (CDC, 2018). In the European Union, it is the second leading cause of food-borne diseases, with *Campylobacter* spp. being the first (EFSA, 2019). Whereas in Brazil, *Salmonella* spp. infections accounted for more than 30% of all food-borne outbreaks between 2000 and 2017 (Brasil. Ministério da Saúde, 2018).

*S.* Heidelberg (*S*H) is among the most prevalent serovars involved in human salmonellosis in North America, Europe, and Brazil (Antunes et al., 2016; Deblais et al., 2018; Duarte, 2019). The incidence of human infections by this serovar increased 25% between 1996 and 2015, even with the decrease of 9% in the total number of serovar-unspecified salmonellosis cases (Foley and Lynne, 2008; Alzwghaibi et al., 2018; Duarte, 2019). In addition, emerging microbial threats such as multi-drug resistant (Parisi et al., 2018; Gupta et al., 2019) and virulent (Giuriatti et al., 2017; Nakao et al., 2018; Yuki et al., 2019) strains have increased the global concern about this serovar. Studies have shown the presence of multi-drug resistance to β-lactam antibiotics such as third-generation cephalosporins (Parisi et al., 2018), along with very invasive strains, causing severe salmonellosis that has progressed to sepsis and endocarditis in identified outbreaks in the United States (Nakao et al., 2018; Yuki et al., 2019).

The molecular mechanisms involved in *S*H virulence are complex and different genes are involved in adhesion (*lpfA*; *agfA*), invasion (*ompC*; *invA*), and colonization (*avrA*). They enable survival and multiplication of the microorganism in host cells, which initiate a series of subsequent events that trigger the disease (Suzuki, 1994). Furthermore, the ability to form biofilms can be regulated by these and other genes, such as those that interfere with the quorum sensing mechanism (*luxS*) and allow communication between bacterial cells (Borges et al., 2018). Together, factors that favor virulence and biofilm formation can create an even more favorable environment for the exchange of plasmid genes (Deblais et al., 2018; Oladeinde et al., 2019), such as those encoding resistance to quinolone (*qnrA*, *qnrB*, and *qnrS*), polymyxins (*mcr*-*1*), and β-lactam (*bla*_TEM_, *bla*_SHV_, *bla*_CTX–M_, and *bla*_CMY–2_) that can express resistance to different classes of antibiotics that intensify the difficulty in treatment in case of infections (Monte et al., 2019). The growing concern about the impact of antimicrobial resistance for a wide range of drugs on animal bacteria of public health importance also includes serovar *S*H. The increased resistance to the β-lactams such as penicillin and cephamycin (Monte et al., 2019), carbapenems (Mthembu et al., 2021) and broad-spectrum cephalosporins (das Neves et al., 2020), as well as to other groups of non-β-lactam antimicrobials such as tetracyclines (das Neves et al., 2020; Souza et al., 2020), quinolones (Monte et al., 2019), sulfonamides (Rodrigues et al., 2020), and polymyxins (Werlang et al., 2018) amplify the severity of clinical cases that require hospitalization, as they are important drugs for therapeutic use.

Brazil is the largest exporter of chicken-based products Associação Brasileira de Proteína Animal (ABPA, 2020), with automated production and strict sanitary control for *Salmonella* spp. (Brasil, 2016; Mouttotou et al., 2017). Despite this, it is important to highlight the increasing number of *S*H isolates in poultry products, a consequence of the increase in the genetic variability of this serotype (Webber et al., 2019). Thus, considering its wide distribution and variety of reservoirs, the different factors that influence the increase in its prevalence, resistance, and virulence must be studied for the effective execution of control measures.

Biofilm formation is an essential factor that has an impact on the contamination of food during its processing. Sessile cells are more resistant than planktonic cells to antimicrobials and typical sanitation processes. Few studies were conducted to characterize biofilms of this serovar (Borges et al., 2018; Silva et al., 2019). Considering the importance and the emergence of *S*H, this study aimed to evaluate the virulence, resistance, and phylogenetic profile, as well as biofilm formation of *S*H strains of poultry origin, discussing their possible danger to public health.

## Materials and Methods

### Sample Collection and Identification

Bacterial isolations were carried out between the years 2017 and 2018 in broiler batches, aged 11–46 days, in eight industrial units (A, B, C, D, E, F, G, and H) that received the animals from five different producers (1, 2, 3, 4, and 5). They were isolated from a Brazilian company, with a complete production cycle and integration system, good standardization and regulatory practices verified by federal inspection, and qualified for national and international trade.

Twenty *S*H strains were analyzed, stored on nutrient agar (NA, Difco), isolated from 17 poultry shed trawl swabs, one fecal sample, one cecum sample and one sample from breast (Table 1). Biochemical identification (ISO, 2002) and serotyping were performed by the Enterobacteria Laboratory of the Oswaldo Cruz Foundation in the state of Rio de Janeiro (IOC/FIOCRUZ, Rio de Janeiro, Brazil).

**TABLE 1:** Data from *S*H strains isolated from Brazilian poultry samples, during the years 2017 and 2018, resistance phenotypic, and genotypic.

Id	Date collection	Company	Producer	Age of animals (days)	Material	Resistance profile	*luxS* and resistance genes (R - *bla*)
H10	02/15/2018	A	1	26	Bed trawl	COL, SMX, TET, AMP, CFT (P3)	*luxS*
H11	02/15/2018	A	1	26	Bed trawl	COL, SMX, TET, AMO, CFT, AMP, CIP (P6)	*luxS/*CMY-2 (R1)
H12	02/15/2018	A	1	26	Bed trawl	COL, SMX, TET, AMO, CFT, AMP (P5)	*luxS/*CMY-2 (R1)
H15	02/16/2018	A	2	27	Bed trawl	COL, SMX, TET, AMO, CFT, AMP, MER (P7)	CMY-2 (R1)
H16	02/16/2018	A	3	26	Bed trawl	COL, SMX, TET, AMP, CFT (P3)	*luxS/*CMY-2 (R1)
H17	02/16/2018	A	4	26	Bed trawl	COL, SMX, TET, AMO, CFT, MER (P4)	*luxS/*CMY-2 (R1)
H18	02/16/2018	A	5	27	Bed trawl	COL, SMX, TET, AMP, CFT (P3)	CMY-2 (R1)
H19	02/16/2018	A	5	27	Bed trawl	COL, SMX, TET, AMP, CFT (P3)	CMY-2 (R1)
H03	02/09/2018	B	1	24	Bed trawl	COL, SMX, TET, AMO, CFT, AMP, MER (P7)	*luxS/*CMY-2 (R1)
H04	02/09/2018	B	2	21	Bed trawl	COL, SMX, TET, AMO, CFT, AMP (P5)	*luxS/*CMY-2 (R1)
H01	02/07/2018	C	1	11	Bed trawl	COL, SMX, TET, AMP, MER (P2)	*luxS/*TEM, CMY-2 (R4)
H02	02/08/2018	D	1	25	Bed trawl	COL, SMX, TET, AMO, CFT, AMP (P5)	*luxS/*CMY-2 (R1)
H05	02/09/2018	D	2	21	Bed trawl	COL, SMX, TET, AMO, CFT, AMP (P5)	*luxS/*CMY-2 (R1)
H06	11/29/2017	E	1	26	Bed trawl	COL, SMX, TET, AMO, CFT, AMP, CIP (P6)	*luxS/*CTX-M (R2)
H07	12/01/2017	F	1	40	Fecal sample	COL, SMX, TET, AMO, CFT, AMP (P5)	*luxS/*CMY-2, CTX-M (R3)
H20	02/16/2018	F	2	44	Bed trawl	COL, SMX, TET, AMO, CFT, AMP, MER (P7)	CMY-2 (R1)
H08	12/23/2017	G	1	46	Cecum	COL, SMX, TET, AMP (P1)	–
H09	02/15/2018	H	1	46	Chest	COL, SMX, TET, AMP, CFT (P3)	*luxS/*CMY-2 (R1)
H13	02/16/2018	H	2	24	Bed trawl	COL, SMX, TET, AMP, CFT (P3)	–
H14	02/16/2018	H	3	34	Bed trawl	COL, SMX, TET, AMO, CFT, AMP (P5)	*luxS/*CMY-2 (R1)
Total N (%)						P1: 1 (5%); P2: 1 (5%); P3: 6 (30%); P4: 1 (5%); P5: 6 (30%); P6: 2 (10%); P7: 3 (15%)	R1: 14 (70%); R2: 1 (5%); R3: 1 (5%); R4: 1 (5%)

*N (%), number and percentage of resistant strains; AMO, amoxicillin with clavulanic acid; COL, colistin; CFT, ceftriaxone; CIP, ciprofloxacin; AMP, ampicillin; MER, meropenem; TET, tetracyclin; SMX, sulfamethoxazole; P, profile; R, *bla*: resistomes described by the *bla* genes.*

### Antimicrobial Susceptibility

The minimum inhibitory concentrations (MICs) were determined for antimicrobials commonly used in human and veterinary medicine. The tests were carried out in triplicates. The tested antimicrobials were ampicillin (AMP), amoxicillin-clavulanate (AMO), colistin (COL), ceftriaxone (CFT), ciprofloxacin (CIP), meropenem (MER), sulfamethoxazole (SUL), and tetracyclin (TET). The test for cefoxitin (30 μg) (FOX) was done by the disk diffusion method, just to complement the discussion. The bacterial inoculum was prepared in NaCl solution 0.9% and the turbidity was adjusted to 0.5 on the McFarland scale (5 × 10^8^ CFU/mL). The suspension of the bacterial inoculum was sown on a Müller Hinton (MH, Difco) agar plate up to 15 min after preparation. The plates were incubated at 35°C for a period of 16–24 h aerobically. The tested concentrations were: 0.5, 1, 2, 4, 8, 16, 32, and 64 mg L^–1^ for all antimicrobials, except for sulfamethoxazole (16, 32, 64, 128, 256, 512, 1,024, and 2,048). The cutoff points were: AMP > 32, AMO > 8/2, COL > 2, CIP > 0.6, CFT > 2, MER > 4, SUL > 512, TET > 16 mg L^–1^ according to CLSI guidelines and recommendations for enterobacteria [Clinical and Laboratory Standards Institute [CLSI], 2019]. Media with the absence of bacteria and *S.* Typhimurium ATCC 14028 were used as sterility control and positive controls, respectively.

### PCR Detection of Virulence and Resistance Genes

Extraction and purification of genomic DNA (gDNA) was performed using the Wizard Genomic DNA Purification Kit (Promega). The quantification of DNA was performed using NanoDrop^TM^ 2000/2000c Spectrophotometer (Thermo Scientific^TM^). PCR reactions were performed using 10 ng of purified DNA. The presence of *ompC* (biosynthesis of outer membrane protein C; invasion), *avrA* (effector protein-colonization), *sodC* (elimination of free radicals), *invA* (invasion), *sefA* (fimbriae-adhesion), *agfA* (fimbriae-biofilm), *lpfA* (fimbriae-adhesion) and *luxS* (quorum-sensing mechanism) genes was determined by PCR. The presence of the genes *bla*_TEM_, *bla*_SHV,_ *bla*_CTX–M_, *bla*_CMY–2_ (linked to beta-lactamases production), *qnrA, qnrB*, and *qnrS* (linked to quinolone resistance), and *mcr-1* (linked to colistin resistance) were also tested in all strains.

PCR reactions were conducted with GoTaq^®^ Green Master Mix kit (Promega), contemplating a final volume of 25 μL. For each reaction, a volume of 12.5 μL of Green Mix, 10.5 μL of MiliQ water, 1 μL of R/F primers (concentrations described in Table 2), and 1 μL of DNA at 10 ng/μL were used. *S.* Enteritidis ATCC 13076 and ultrapure water were used as positive and negative controls, respectively, for the analysis of virulence genes. A *Klebsiella pneumoniae* strain, previously tested, provided by the Molecular Microbiology Laboratory of the Federal University of Uberlândia was used as a positive control, for the presence of antimicrobial resistance genes.

**TABLE 2:** *Primers* used to identify specific genes in *S*H strains.

Gene	Concentration	Amplicon (bp)	Primer 5′–3′	T. annealing	Reference
*ompC*	10 pmol	204	ATCGCTGACTTATGCAATCG CGGGTTGCGTTATAGGTCTG	58°C/30 s	Jawad and Al-Charrakh, 2016
*avrA*	20 pmol	385	GTTATGGACGGAACGACATCGG ATTCTGCTTCCCGCCGCC	62°C/30 s	Prager et al., 2003
*sodC*	20 pmol	500	ATGAAGCGATTAAGTTTAGCGATGG TTTAATGACTCCGCAGGCGTAACGC	62°C/30 s	Sanjay et al., 2010
*invA*	10 pmol	284	GTGAAATTATCGCCACGTTCGGGCAA TCATCGCACCGTCAAAGGAACC	58°C/30 s	Oliveira et al., 2002
*sef*A	10 pmol	488	GATACTGCTGAACGTAGAAGG GCGTAAATCAGGATCTGCAGTAGC	50°C/30 s	Oliveira et al., 2002
*agf*A	10 pmol	350	TCCACAATGGGGCGGCGGCG CCTGACGCACCATTACGCTG	66°C/30 s	Collinson et al., 1993
*lpfA*	10 pmol	250	CTTTCGCTGCTGAATCTGGT CAGTGTTAACAGAAACCAGT	50°C/30 s	Webber et al., 2019
*luxS*	20 pmol	1080	GATAATCCTGAACTAAGCTTCTCCGC GGTTATGAGAAAAGCATGCACCGATCA	62°C/30 s	Choi et al., 2007
*bla*_*TEM*_	10 pmol	643	CAGCGGTAAGATCCTTGAGA ACTCCCCGTCGTGTAGATAA	50°C/45 s	Chen et al., 2004
*blaSHV*	10 pmol	714	GGCCGCGTAGGCATGATAGA CCCGGCGATTTGCTGATTTC	56°C/45 s	Chen et al., 2004
*bla*_*CMY–2*_	10 pmol	870	TGGCCGTTGCCGTTATCTAC CCCGTTTTATGCACCCATGA	59°C/53 s	Chen et al., 2004
*bla*_*CTX–M*_	10 pmol	593	TGGGTRAARTARGTSACCAGAAYCAGCGG CCCCCGCTTATAGAGCAACAACAA	58°C/60 s	Monstein et al., 2007
*Mcr-1*	10 pmol	309	CGGTCAGTCCGTTTGTTC CTTGGTCGGTCTGTAGGG	58°C/40 s	Liu et al., 2016
*qnrA*	10 pmol	580	AGAGGATTTCTCACGCCAGG TGCCAGGCACAGATCTTGAC	54°C/60 s	Cattoir et al., 2007
*qnrS*	10 pmol	428	GCAAGTTCATTGAACAGGGT TCTAAACCGTCGAGTTCGGCG	54°C/60 s	Cattoir et al., 2007
qnrB*	10 pmol	264	GGMATHGAAATTCGCCACT TTTGCYGYYCGCCAGTCGA	54°C/60 s	Cattoir et al., 2007

T, temperature.*Able to amplify the *qnrB1* to *qnrB6* variants.

Amplification was performed in a thermocycler (Eppendorf^®^), with an initial denaturation cycle at 94°C for 5 min and 35 amplification cycles: denaturation at 94°C for 45 s, annealing according to Table 2, extension at 72°C for 90 s, with final extension at 72°C for 10 min. The amplification conditions for the antimicrobial resistance genes to β-lactams differed in terms of the number of cycles (30) and annealing conditions (Table 2). For the *qnrA, qnrB*, and *qnrS* genes the initial denaturation was 95°C for 10 min, followed by 35 denaturation cycles at 95°C for 1 min, annealing (Table 2), extension at 72°C for 90 s, and finally, final extension at 72°C for 10 min. The amplified products were submitted to electrophoresis in 1.5% agarose gel, using the TBE 0.5x running buffer (Invitrogen) and as a molecular weight standard, the 100 bp marker (Invitrogen).

### Genotyping by Pulsed-Field Gel Electrophoresis

To verify the genetic relatedness of isolates, pulsed-field gel electrophoresis (PFGE) analysis was executed using the PulseNet protocol, as previously recommended (CDC, 2017). Bacteria grown at 37°C overnight on Tryptic Soy Agar (TSA) (OXOID^®^) were suspended in tubes containing 2 mL of phosphate buffered saline (PBS: 0.01 M phosphate buffer; pH 7.2; 0.85% NaCl). After agarose blocking, gDNA digestion was performed with 30 U of *Xba1* enzyme (Invitrogen^®^) for 2 h at 37°C.

The DNA fragments were separated on 1% agarose gel (SeaKem Gold^®^) in 0.5X TBE buffer in CHEF DRIII (Bio-Rad^®^, California, United States) for 18 h with the following parameters: 200 V, 120° angle, 6 V/cm gradient and 14°C buffer temperature. The gels were stained with ethidium bromide, photographed under UV light in a transilluminator (Loccus Biotechnology^®^) and evaluated using the BioNumerics program.

### Biofilm Production

**FIGURE 1:**
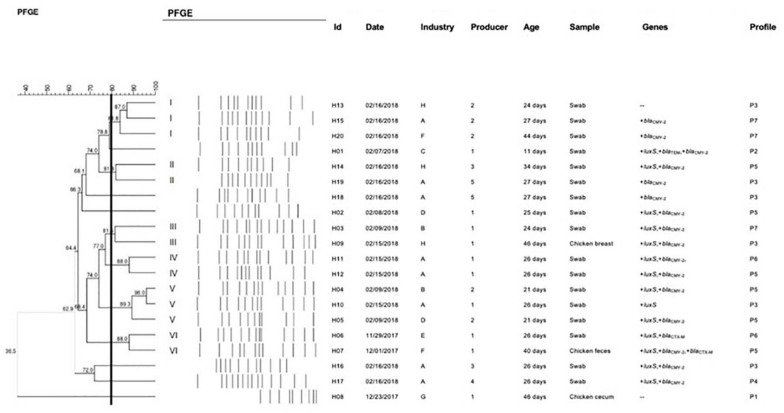
Comparative dendrogram of 20 *S*H strains, constructed from PFGE results considering the isolation site, date of collection, and the presence or absence of specific genes and antimicrobial resistance, using the Dice similarity coefficient with 1.5% tolerance and UPGMA method with 0.80% optimization. I-VI, pulsotypes; Id, Identification. Genes: only those that showed differences between the strains. Profile: antimicrobial resistance profile (P1-P7; Table 1).

Qualitative and quantitative phenotypic analyzes of the sessile structure at temperatures of 4, 25, and 37°C were performed using two phylogenetically different strains, from different origins and with differing virulence and resistance profiles (H06 and H18 – Table 1 and Figure 1). To evaluate the cell adherence of the selected strains, bacterial inoculum (10^4^ CFU/mL–OD_600_ = 0.22–0.28) was centrifuged (5,000 rpm, 10 min, 4°C) and washed twice (NaCl 0.9%). Then, 20 mL of Tryptic Soy Broth (TSB) (Merck^®^) was added, and in parallel, 20 mL of TSB was supplemented with 5% chicken juice (CJ) to simulate industry conditions and compare both situations (Brown et al., 2014).

Qualitative analysis of the biofilms was performed according to recommendations (Kudirkienë et al., 2012), with modifications, including eight replicates in each of the three repetitions. Briefly, 200 μL of the bacterial suspension in TSB medium and TSB enriched with CJ was added in 96-well plates. For biomass formation, the plates were incubated for 24 h at different temperatures (4, 25, and 37°C) under agitation (100 rpm). After incubation, the washed and dried total biomass was measured by fixing with 0.1% Violet Crystal (LaborClin), followed by elution with alcohol-acetone solution (80:20 v/v ethanol-acetone) (Dinamica^®^).

The determination of the biofilm formation index (BFI) was carried out according to Stepanović et al. (2000) from the results of reading the OD_600_. As cutoff (ODc), the average value of OD_600_ equivalent of 0.044, obtained by adding the average optical density of negative controls (which is 0.007) the offset value of the standard multiplied by three (0.0124 × 3), was used. The classification of biofilms was determined as follows: (a) non-existent: OD_600_ ≤ 0.044 (less than ODc); (b) weak: 0.088 ≤ OD_600_ > 0.044 (up to two times higher than ODc); (c) moderate: 0.176 ≤ OD_600_ > 0.088 (between two to four times the ODc value); and (d) strong: OD_600_ > 0.176 (greater than four times the ODc value).

Quantitative adhesion (2 h of incubation) and biofilm tests (24 h of incubation) were determined by the number of CFUs of sessile cells. Biofilms were formed under the same conditions described above for the qualitative test with three replicates per repetition. After the formation of the adhered mass and biofilm, the wells were washed twice with 0.9% NaCl solution and the biomass was removed by scraping the wells for 90 s. The cell suspension obtained was subjected to serial dilutions and seeded on TSA agar plates to obtain the CFU number.

### Scanning Electron Microscopy

The ultrastructure of the sessile form of H06 and H18 was evaluated by Scanning Electron Microscope (SEM), using a modified method followed by Brown et al. (2014). Biofilms were formed in glass spheres with a diameter of 5 mm, in TSB respecting the growth conditions described above. After biomass formation, the samples were fixed with 2.5% glutaraldehyde and 2.5% paraformaldehyde in 0.1 M PBS buffer (pH 7.4) overnight at 4°C. Samples were washed three times with PBS buffer. The beads were post-fixed with 1% osmium tetroxide for 1 h and washed three times with PBS buffer. The spheres were dehydrated in a series of ethanol solutions (30, 40, 50, 60, 70, 80, and 90% and then three times at 100%) for 15 min in each step.

The samples were dried on CPD (Critical Point Drying) (CPD 030, Baltec, Germany) using liquid carbon dioxide as the transition fluid, then coated with a 20 nm thick gold layer (SCD 050, Baltec, Germany) and visualized in MEV VP Zeiss Supra 55 FEG SEM operating at 20 kV.

### Biofilm Inhibition Assay

To examine the interaction between H06 and H18 biofilms with disinfectant components (0.8% peracetic acid, 1% sodium hypochlorite and 1% chlorhexidine), a modified protocol followed by Nguyen and Yuk (2013) was used.

An aliquot of 100 μL (10^7^ cells) was inoculated on the surface of a sterile cellulose membrane with porosity of 0.45 μm and 47 mm in diameter, on a TSA plate (Merck^®^). The plates were incubated at 37°C and had the membrane transferred to a new plate every 24 h for 3 days. Subsequently, the membrane was placed in a flask containing 20 mL of TSB broth with the concentrations of the disinfectant components. After incubation at 37°C for 15 min, the membrane was washed three times with phosphate buffer (PBS), followed by treatment in 25 mL of 0.1% trypsin for 15 min at room temperature. Then, the resulting solution underwent serial dilutions for subsequent counting on TSA plates, at 37°C for 24 h.

### Genomic Sequencing

The strains H06 and H18 had their genomes sequenced. First, gDNA was obtained through Wizard Genomic DNA Purification Kit (Promega). A 450 bp read library was constructed using NEBNext Fast DNA Fragmentation and Library Preparation Kit (New England Biolabs, Ipswich, NE, United States) and quality checked by the Agilent 2100 Bioanalyzer.

The gDNA library was then sequenced by 2 × 150 bp paired-end sequencing on an Illumina HiSeq 2500 sequencing platform (Illumina, San Diego, CA, United States). Read quality was assessed using FastQC (Andrews, 2010). *De novo* assembly was performed using Unicycler (v3.1) (Wick et al., 2017). Assembly quality statistics were generated using QUAST (Gurevich et al., 2013). Initial scaffolding was performed using SSPACE (Boetzer et al., 2011) and the gaps closed using GapFiller (Nadalin et al., 2012). *S.* Heidelberg SH14-009 genome sequence obtained from GenBank (accession CP016581.1) was used as the reference to generate final scaffold in CONTIGuator (Galardini et al., 2008). Genome completeness was assessed using Benchmarking Universal Single-Copy Orthologs (BUSCO) v4.0.6 (Simao et al., 2015). The presence of plasmid-predicted contigs was accessed using gplas (Arredondo-Alonso et al., 2020). Its results, along with completed circular contigs predicted by Unicycler, were merged to obtain all predicted plasmid sequences (circular and uncircular contigs). Then, BLASTn against the non-redundant database of NCBI was used to confirm the plasmid prediction. Identified plasmids had their sequences extracted and the remaining contig sequences were considered part of the bacterial chromosome. Annotation was performed using Prokka (Seemann, 2014). The sequenced *S*H genomes were deposited at the DDBJ/ENA/GenBank under the accession numbers JAGFJR000000000 (H06) and JAGEOP000000000 (H18).

### *In silico* Genomic Characterization

ABRicate (v 0.9.9) (Seemann, 2020) was used to screen virulence and resistance genes. This tool allows the use of multiple databases. Search for virulence genes was completed using the VFDB database (Chen et al., 2016). Resistance genes were screened using the CARD database (Jia et al., 2017). The VFDB database includes 2,597 curated genes related to virulence factors whereas the CARD database includes 2,617 genes related to antimicrobial resistance. Hits were only considered for gene lengths ≥75% and identities of ≥75%.

Presence of chromosomal mutations was predicted using ResFinder v. 4.1 with default settings (90% selected percentage identity threshold and 60% selected minimum coverage length), available at the Centre for Genomic Epidemiology^1^.

Virulence genes related to biofilm formation selected by manual curation was used as input to generate an association protein-protein network in STRING (Szklarczyk et al., 2019). Pathways significantly enriched in KEGG PATHWAY database^2^ were then checked.

### Data Analyses

In addition to descriptive statistics, the quantitative assays with biofilms tests were compared by one-way analysis of variance (One-way ANOVA). Two variables’ comparisons were performed using student’s *t*-test and Fisher exact test. Confidence level of 95% was considered, using the GraphPad Prism software, version 8.0 (GraphPad Software, United States).

Analysis for dendrogram construction was conducted using BioNumerics software. The comparison of the band patterns was performed by the UPGMA analysis method, using the Dice similarity coefficient with a tolerance of 1.5% in the comparison of the position of the bands.

## Results

### Virulence Factors and Resistance Profiles

All strains (100%) of the study carried the *ompC*, *invA*, *sodC*, *avrA*, *lpfA*, and *agfA* genes, but only 70% (14/20) of them had the *luxS* gene. No strains showed the presence of the *sefA* gene. Table 3 shows the percentage of strains with phenotypic resistance to the tested drugs, demonstrating that the highest susceptibility (*p* < 0.05) was identified for CIP (90%), MER (75%), and AMO (40%). All strains were resistant to COL, TET, and SMX, being characterized as multidrug resistant (MDR) strains.

**TABLE 3:** Distribution of MIC and resistance index of *S*H.

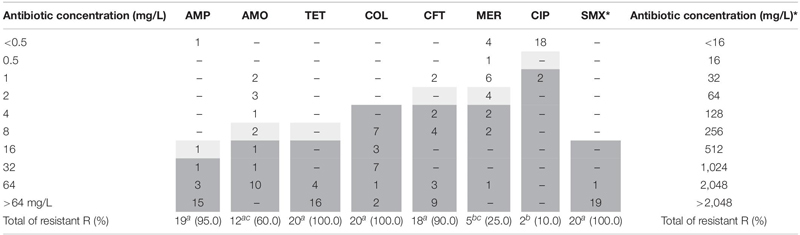

*AMO, amoxicillin with clavulanic acid; COL, colistin; CFT, ceftriaxone; CIP, ciprofloxacin; AMP, ampicillin; MER, meropenem; TET, tetracyclin; SMX, sulfamethoxazole; ___ (line), cutoff point according to Clinical and Laboratory Standards Institute [CLSI], 2019; dark gray, resistant strains; light gray, strains with intermediate resistance; R (%), resistance index.*

Different letters on the line indicate a significant difference (Fisher’s Test *p* < 0.05).

*Specific concentrations and results for the sulfamethoxazole test (SMX).

The identified resistance profiles (Table 1) show resistance to at least four classes of antimicrobials. 60% (12/20) of the strains showed resistance to at least six drugs and include profiles P4, P5, P6, and P7. The resistome analysis did not identify the presence of the *bla*_*SHV*_, *qnrA, qnrB*, and *qnrS* genes in any of the strains. However, all of them showed phenotypic resistance to the classes of penicillin (AMP and AMO) and/or cefens (CFT, FOX). This result was consistent with the genotype (R1 to R4) related to the presence of one or more of the *bla* genes tested in 17 (85%) from them.

The presence of *bla* genes co-produced in this group was identified in 2/17 (11.8%), corresponding to the R3 and R4 resistomes, determinants of the phenotypic resistance to penicillins (R4), and to the cefens in R3. For R2 and R3 resistomes showed the presence of the *bla*_*CTX–M*_ gene found in 2/20 (10%), expressing phenotypic resistance to penicillin and CFT. Only one strain (1/20–5.0%) (R4) presented the *bla*_*TEM*_ gene and expressed phenotypic resistance to ampicillin in the MIC test.

The *bla*_*CMY–2*_ gene was the most prevalent (16/20 – 80%) and was identified in the R1 and R3 resistomes. In addition to determining phenotypic penicillin resistance, unanimous resistance to CFT was observed. In parallel, disc diffusion assay showed that the four strains resistant to cefoxitin (FOX) presented this gene in their resistome (data not shown).

### Clustering of Strains

The dendrogram constructed from the PFGE results was compared considering the isolation site, date of collection, and genotypic and phenotypic characteristics evaluated. Similarity analysis of the 20 strains of *S*H presented six pulsotypes (I – VI; Figure 1) that showed genotypic similarities above 80%. In opposition, six profiles showed little genetic proximity (<80%).

Strains isolated from different years were not grouped in the same pulsotype. The same idea applies to the producer (except for pulsotypes II and V) and the gene panel (except for pulsotype III and IV). The other parameters were not decisive for clusters discrimination.

### Characterization of *S*H Biofilms

Strains H06 and H18 demonstrated the same ability to adhere when inoculated in TSB and TSB + 5% CJ (*p* = 0.153). Considering the initial inoculum of approximately 10^3^ CFU/well, it was found that there was an increase in counts after the adhesion process (*p* < 0.05) at temperatures of 25 and 37°C, but the presence of CJ did not favor this process at the quantitative level (*p* = 0.124) (Table 4).

**TABLE 4:** Mean counts (Log CFU/mL) and BFI (Biofilm Formation Index) obtained in the tests for the two strains of *S*H under different temperature conditions.

Test	Strain	Media	Initial inoculum	4°C		25°C		37°C
Adhesion	Both mean	TSB	3.22 ± 0.29 a	3.99 ± 0.21 a		4.05 ± 0.12 b		4.81 ± 0.33 b
		TSB + CJ	3.25 ± 0.32 a	3.74 ± 0.12 a		4.64 ± 0.25 b		4.86 ± 0.43 b
Biofilm	Both mean	TSB	3.22 ± 0.29 a	4.895 ± 0.35 b		5.825 ± 0.51 c		6.471 ± 0.49 c
		TSB + CJ	3.25 ± 0.32 a	4.456 ± 0.5 b		6.294 ± 0,0.3 c		6.529 ± 0.41 c
Biofilm	H06	TSB	–	5.130 ± 0.14* a	I	6.373 ± 0.19* b	I	7.256 ± 0.02* b
		TSB + CJ	–	4.485 ± 0.38* a		7.274 ± 0.03* b		7.206 ± 0.01* b
Biofilm	H18	TSB	–	4.731 ± 0.36 a	I	5.459 ± 0.22 b	I	6.320 ± 0.10 b
		TSB + CJ	–	4.436 ± 0.35 a		5.263 ± 0.06 b		6.452 ± 0.01 b
BFI (Class.)	Both mean	TSB	–	0.0353 (N)		0.1611 (M)		0.1622 (M)
		TSB + CJ	–	0.0329 (N)		0.3491 (S)		0.3970 (S)

*Class., classification by BFI; N, non-existent; M, moderate; S, strong.*

Different letters indicate significant difference compared to the initial inoculum, the *test* one-way ANOVA (adhesion test). Different letters indicate significant difference, one-way ANOVA test (biofilm test).

*Indicate significant when comparing the culture media TSB and TSB + CJ, student’s *t* test (biofilm test in individual strain). Iindicate significant difference when strains are compared in relation to temperature, student’s *t* test (biofilm test in compare strains).

The 2-h incubation period was sufficient for the initial establishment of the biofilm structure, except for the temperature of 4°C, which allows us to infer that lower temperatures may help in the initial control of the formation of the sessile structure in *S*H. At 4°C, the presence of biofilm was not verified by the Biofilm Formation Index (BFI). Both strains showed increased capacity to form biofilms linked to the temperatures 25 and 37°C, intensified after supplementation with CJ that allowed the production of strong biomass.

Individually, from a constant initial inoculum (*p* > 0.05) in all assays, it was observed that for the strain H06 there was a significant increase in TSB + CJ counts when compared to TSB counts at 25°C, but the reverse was observed at 4 and 37°C. For both strains, 4°C was the temperature that least favored bacterial multiplication. More efficient replication capacity in the sessile form, considering the conditions supplemented or not with CJ, was observed at 25 and 37°C for strain H06 (Table 4).

The joint evaluation of the strains demonstrated that the CJ did not influence the number of bacteria present in the biofilm at the three temperatures tested, but favored the greater production of extracellular matrix at 25 and 37°C. Similarly, the increase in temperature from 4 to 25°C or 37°C increased (*p* < 0.05) the number of bacteria adhered in biofilms (Table 4).

**FIGURE 2:**
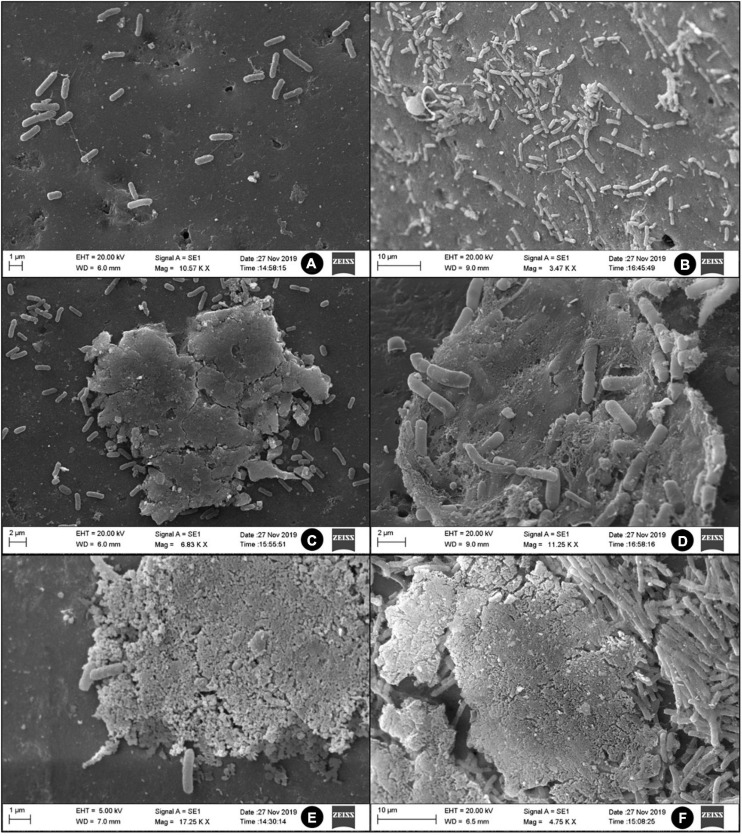
SEM images of two strains of *S*H at temperatures of 4 **(A,B)**, 25 **(C,D)**, and 37°C **(E,F)** in TSB.

SEM showed changes in the biomass formed in both strains cultivated in TSB at different temperatures (Figure 2). Figures 2A,B show a minimum synthesis of extracellular matrix fibers in isolated bacteria, indicating the incapacity to form biofilm at 4°C. This result is consistent with the qualitative assay found in the biofilms. In Figures 2C,D, the initial biofilm formation were observed with more intense extracellular matrix production at 25°C, while in Figures 2E,F the presence of a three-dimensional structure of the matrix becomes more evident, indicating the development of a mature biofilm at 37°C.

**FIGURE 3:**
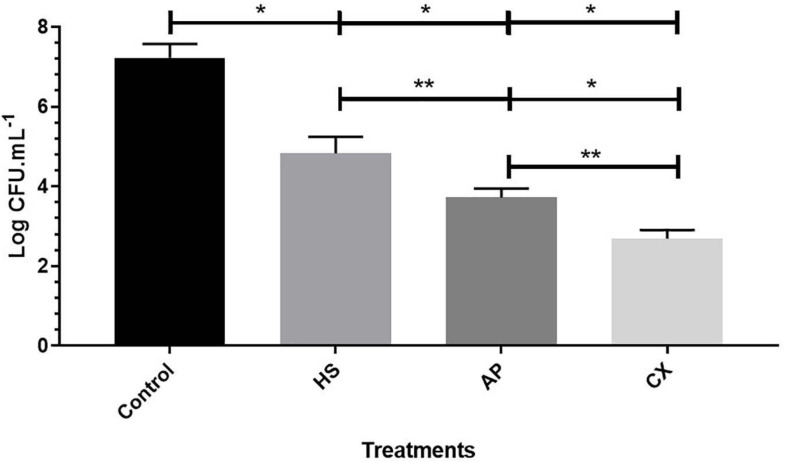
Graph of biofilm counts for both strains of *S*H (log of CFU mL^−1^) in control and maintained for 15 min in 0.8% peracetic acid (AP), 1% sodium hypochlorite (HS) and 1% chlorhexidine (CX). *p < 0.0001; **p < 0.001 using one-way ANOVA for counts between treatments.

Greater biomass production was observed at 25 and 37°C in both strains. The characteristic of the matrix in these biofilms indicates a dense and compact structure. However, the inhibition of biofilm formation by chemical agents demonstrated the same behavior in both strains for all treatments (*p* > 0.2). None of the treatments promoted total elimination of viable cells, but all agents significantly reduced the counts (Figure 3) of *S*H in relation to the untreated biofilm (7.23 log CFU/mL).

The treatment with sodium hypochlorite 1% reduced the number of sessile bacteria to 4.83 log CFU/mL, while peracetic acid 0.8% reduced about 3.51 log cycles compared to untreated biofilm. The use of chlorhexidine 1% had the greatest effect on the control of the sessile structure with a viable biomass of 2.69 log CFU/mL, representing a reduction of 4.53 log cycles.

### Genome Overview and *in silico* Prediction

**FIGURE 4:**
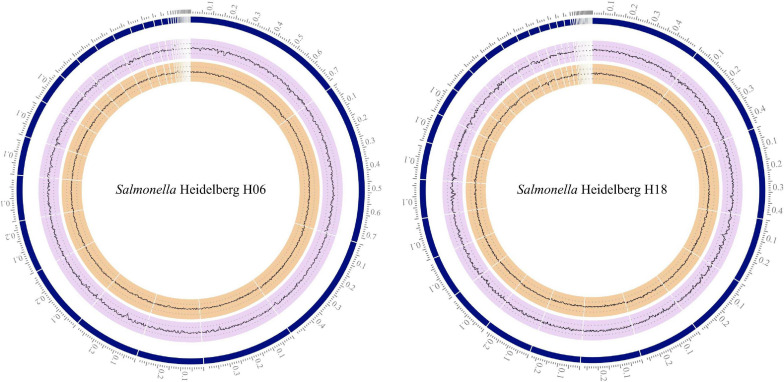
Circular graphical display of the distribution of the contigs of *Salmonella* Heidelberg H06 and H18 genomes assembly. From outer to inner rings, we present contigs (in Mbp), GC content, and GC skew, produced by Circos software.

The assembled chromosome of strain H06 had 35 contigs, with the total length of 4,731,348 bp and an average G + C content of 52.17%, while strain H18 had 43 contigs, with the total length of 4,838,250 bp and an average G + C content of 52.11% (Figure 4). Findings of BUSCO analysis in the genomes of strains H06 and H18 showed 99.5 and 99.5% completeness, respectively, using the *Enterobacterales* dataset based on conserved orthologous genes among species within the order.

Gene prediction and annotation of the *Salmonella* strains resulted in 4,431 and 4,544 predicted protein coding sequences (CDS), for strains H06 and H18, respectively, 76 transfer RNA (tRNA) genes for both strains, and four ribosomal RNA (rRNA) genes in strain H06, while five in H18.

Plasmid prediction of the assembled contigs of *S*H genomes resulted in three plasmids on strain H06 and six on strain H18.

**FIGURE 5:**
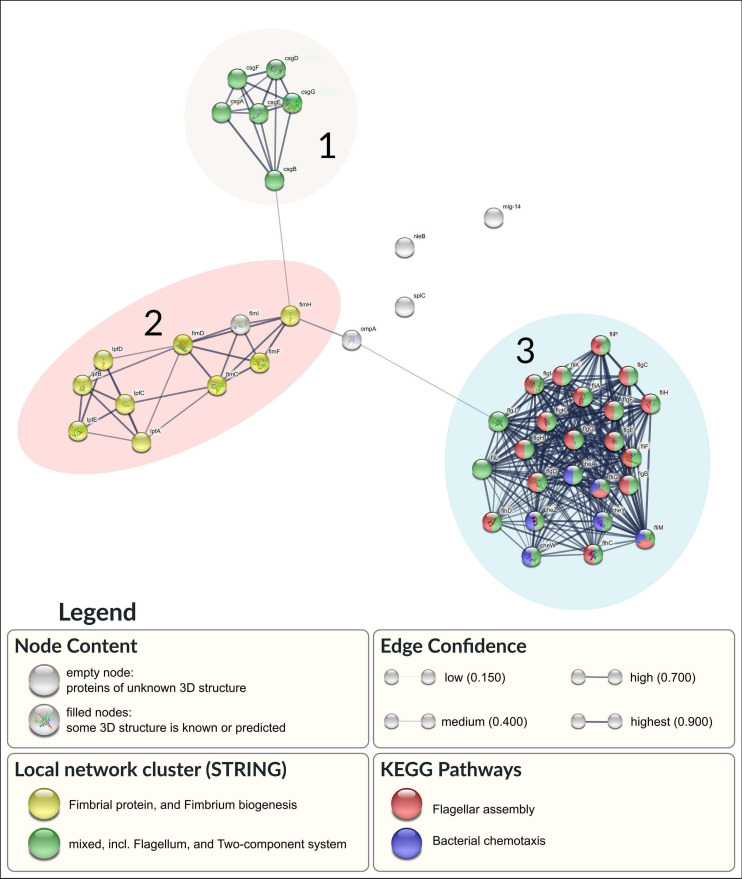
Association network in STRING of the 44 selected proteins. Network nodes represent proteins. Proteins with enriched pathways are colored by red (Flagellar assembly) and blue (Bacterial chemotaxis). Additional node clustering is highlighted in yellow (Fimbria biogenesis) and green (Flagellum and Two-component system). Edges represent predicted protein-protein association with specific confidence score, according to its thickness.

The results of the genomic virulence typing for both isolates are shown in Supplementary Table 1. Among the two isolates, 135 different virulence genes were identified. The majority of the genes (127/135) were present in both genomes. Only eight genes were exclusively found in the genome of strain H18. All of them (*sptP*, *prgH*, *prgI*, *prgJ*, *prgK*, *orgA*, *orgB*, and *orgC*) code for proteins related to a type III secretion system. Manual screening for virulence genes coding for proteins related to biofilm formation resulted in 44 proteins (Supplementary Table 2). Interactions between them are shown in Figure 5.

A total of 49 different resistance genes were identified, belonging to 24 classes of antibiotics. Five of these genes [ANT (3″)-IIa, AAC (3)-Via, *sul1*, *bla*_*CTX–M–2*_, and *bla*_*CMY–59*_] are strain specific (the first four in H06 and the last one in H18) (Supplementary Table 3). The presence of plasmid genes associated with resistance to aminoglycosides (*aac3*, *aac6*, and *ant3*), sulfonamides (*sul1* and *sul2*), β-lactams (*bla*_*CTX–M–2*_ and *bla*_*CMY–2*_), fosfomycin (*fosA7*), and tetracycline (*tetA*), featuring an alarming multi-resistance profile.

The gene *bla*_*CTX–M–2*_ was found only in the genome of the strain 6H, while *bla*_*CMY–2*_ only appeared in the strain 18H. None of the strains carries the *qnrB* gene. Point mutations in DNA *gyrA*se subunit A (*gyrA*; Ser83Phe) and DNA topoisomerase IV subunit C (*parC*; Thr57Ser) conferring resistance to nalidixic acid and ciprofloxacin were observed in both strains. No mutation was detected in *gyrA*se subunit B (*gyrB*) and topoisomerase IV subunit E (*parE*). Mutation in *gyrA* occurred at the eighty-third codon, where the amino acid serine was substituted by phenylalanine (*gyrA*; Ser83Phe). Mutation in *parC* occurred at the fifty-seventh codon, where the amino acid threonine was substituted by serine (*parC;* Thr57Ser).

## Discussion

### Antimicrobial Resistance Profile

Our findings demonstrated higher phenotypic antimicrobial resistance against to AMO, AMP, COL, CFT, SMX, and TET commonly used in veterinary and human medicine routine when compared to results obtained in Brazil and Argentina (Borsoi et al., 2009; Aravena et al., 2019).

There are no reports of studies in Brazil on resistance to colistin in *S*H in birds, but the previous permission to use this drug in poultry production generated a selection pressure of resistant strains that remained, even after the ban of its use in 2016 (BRASIL, 2016). Other serovars with this phenotype have been identified and associated with the presence of the plasmid-mediated *mcr* gene (Borowiak et al., 2019; Trujillo-Soto et al., 2019) that codes for phosphoethanolamine transferase (pEtN transferase) and changes the LPS, target of this antibiotic, inhibiting the action of colistin (Baron et al., 2016). However, this gene was not detected in our strains, the *ugd* gene was identified in the H06 and H18 strains by WSG, and may also be the cause of the observed phenotype since it encodes polymyxin resistance (Mouslim and Groisman, 2003).

Similar to colistin resistance, 90% of the studied strains were resistant to third-generation cephalosporins, in particular for ceftriaxone, an important drug used to treat children with salmonellosis (das Neves et al., 2020). The presence of the *bla*_*CMY–2*_ gene is consistent with this phenotype, due to the overproduction of AmpC induced by the presence of cephalosporin and cephamycin that exhibit resistance to extended-spectrum cephalosporins, such as ceftriaxone, and to cefoxitin (identified in four of the 20 strains – data not shown) (De Angelis et al., 2020). The high prevalence of *S*H AmpC strains highlights the endemic occurrence of β-lactam resistance in North and South America (Edirmanasinghe et al., 2017; Monte et al., 2019; van den Berg et al., 2019; Souza et al., 2020).

β-lactam antibiotics act on the bacterial cell wall, interfering with the synthesis of the peptidoglycan layer. Bacterial resistance mediated by plasmids belonging to the *bla* subgroups, such as *bla*_*CTX–M*_, is highlighted by their ability to produce extended-spectrum β-lactamases (ESBLs). Considering the importance of this antibiotic class as the first-choice treatment of salmonellosis, the presence of resistant strains is alarming. It is estimated that approximately 26,000 infections and 1,700 deaths occur annually in the United States caused by ESBL-producing strains, generating enormous hospital expenses to the country. Recently, the number of cases reporting ESBL-producing *Salmonella* has increased worldwide, becoming one of the major public health concerns. *bla*_*CTX*_ and *bla*_*CMY–2*_ are the most identified genes in isolates of food-producing animals (Biffi et al., 2014; Qiao et al., 2017; Jeon et al., 2019).

The four identified resistomes (Table 1) revealed a high level of resistance to amoxicillin, ampicillin and/or ceftriaxone. Although the *bla*_*CTX–M*_ gene is reported with a higher incidence of *Salmonella* in poultry products (Moura et al., 2018; Perin et al., 2020), our study shows that its frequency was low (2/20–10%) and corroborates with recent studies carried out with samples Brazilian samples (van den Berg et al., 2019; Souza et al., 2020). Gram-negative ESBL-producing and hyper-producing AmpC bacteria have also stood out for their resistance to carbapenems, such as meropenem, identified in 20% (4/20) of our strains, when associated with other mechanisms, such as hyper expressed efflux systems (Andrade and Darini, 2017).

The phenotypic co-resistance to TET and SUL identified in all strains in the present study may be associated with the expression of *tetA* as well as the *sul1* and *sul2* genes, detected in strains H06 and H18 by WSG. *tetA* gene, like the *tet* gene complex, has wide distribution in several serovars of *Salmonella* (Michael et al., 2006; Delgado-Suárez et al., 2019), including *S*H (Keefer et al., 2019). Tetracyclines have a great capacity for intracellular diffusion, reversibly preventing protein synthesis (Brasil. Ministério da Saúde, 2007; Luiz et al., 2008). The *sul1* and *sul2* genes encode forms of enzymes (dihydropteroate synthase) that are not inhibited by sulfonamides (Lynne et al., 2008). In parallel, we also identified the plasmid pSH-04-1 in H06 and H18, which was associated with resistance to sulfonamides, β-lactams, aminoglycosides, and tetracycline in *S*H (Hoffmann et al., 2014; Keefer et al., 2019).

Although we did not identify *qnr* in any of the strains, nor *via* WSG, we can associate the resistance of 2/20 (10%) to this drug with the presence of point mutations in *gyrA* at codons Ser83Phe and parC; Thr57Ser, in H06, that conferring phenotypic resistance to ciprofloxacin. The same mutation regions that confer resistance to quinolones have also been identified by Monte et al. (2019) in *S*H.

In addition to the genes already mentioned, four of them (*aac3, aac6, ant3*, and *fosA7*) were also previously reported in *S*H strains isolated from clinical, environmental and animal samples (Hoffmann et al., 2014; Keefer et al., 2019) with high potential for plasmid gene transfer, *via* conjugation, in species of the order *Enterobacterales* in the intestinal microbiota (Lim et al., 2013).

The *aac3, aac6*, and *ant3* genes are directly involved in resistance to aminoglycosides and acts to alter the function of the bacterial ribosome by binding to its 30S fraction, inhibiting protein synthesis or producing defective proteins. The identified resistance plasmids encode proteins that act in enzymatic modifications of antibiotics, mainly gentamicin (Brasil. Ministério da Saúde, 2007; Luiz et al., 2008; Noh et al., 2019).

The presence of *fosA7* (recently reported – 2017) is alarming because its sequence was found in only 36 of the approximately 40,000 genomes in the NCBI database. 75% of them belong to serovar *S*H. In addition, studies show that this gene easily integrates with *Salmonella* chromosomal DNA (Keefer et al., 2019; Rehman et al., 2019). Fosfomycin is used to treat systemic diseases caused by members of the *Enterobacteriaceae* family with advanced resistance to antimicrobial drugs, including ESBL producers (Hou et al., 2012). The presence of the *fosA7* gene was identified *via* WGS, co-existing with *bla*_*CTX–M*_ (strain H06) and *bla*_*CMY–2*_ (strain H18). There are other reports that identified *fosfA7*/*bla*_*CMY–2*_ in *S*H (Rehman et al., 2019) and *fosA3*/*bla*_*CMY–2*_ in other enterobacteria (Zhao et al., 2015). This shows that these genes can be co-selected and transported in plasmids IncFIIa and F33: A-: B- for *bla*_*CTX–M*_ (Hou et al., 2012; Cunha et al., 2017) and HD0149 for *bla*_*CMY–2*_ blaCMY-2 (Zhao et al., 2015), intensifying the horizontal transfer of genes and MDR profile in *S*H (Rehman et al., 2017) and other enterobacteria (Zhao et al., 2015).

The MDR profile identified in all strains intensifies the public health alert, since there was an outbreak involving multidrug-resistant *S*H in the United States (Nakao et al., 2018). Between January 2015 to November 2017, 56 people were infected in 15 United States’ states by multidrug-resistant *S*H strains (β-lactams, fluoroquinolones, tetracyclines, aminoglycosides, sulfonamides, and carbapenems), from veterinary environments (CDC, 2018). In Brazil, similar results were found by other researchers, with resistance in *S*H to different antibiotic classes, such as penicillins, cephalosporins, quinolones, streptomycin, nalidixic acid, cefotaxime, cefoxitin, ceftiofur, and tetracyclines (Monte et al., 2019; Souza et al., 2020).

The identification of five strains (25%) resistant to seven tested drugs (P6 and P7) indicates the indiscriminate use of antimicrobials and neglect of methods of preventing biofilm formation, which facilitates recombination and the exchange of genes linked to the resistance and expression of efflux pumps (Mouttotou et al., 2017). The current emergence of *S*H and its dissemination in Brazil has demonstrated critical data related to its microevolution and the implementation of control measures (Voss-Rech et al., 2019). In general, the presence of (i) resistance genes acquired *via* recombination, (ii) efflux pumps (with 27 genes identified in H06 and H18) and permeability barrier (*pmrF*, present in H06 e H18), and (iii) exposure to sublethal doses as in metaphylaxis procedures are commonly associated to the appearance of multi-drug resistance (MDR) in the serovar *S*H (Deblais et al., 2018).

To assess the overall prevalence of clinical and environmental isolates of *S. enterica* harboring the identified plasmids, data from the NCBI – Pathogen detection database^3^ were analyzed. Among the 205,032 genomes of *Salmonella enterica* selected, only 0.4–21.1% showed at least one of these genes. However, the serovar *S*H showed the highest prevalence of these genes (64.4%). Among all, 58.2% are environmental and chicken-isolated samples, while only 6.2% are described as clinical isolates (Rehman et al., 2019).

### Specific Virulence Genes

Previous studies with *S*H isolated from various sources between 1985 and 2011, from the United States and Brazil, shows that virulence factors are highly conserved for this serovar (Hoffmann et al., 2014). Our results are consistent with these findings, as 6/8 genes were identified in all strains.

The *ompC* and *invA* genes are used to characterize the genus and the ability to invade host tissues, respectively, and the presence in all strains was expected. Together with the *agfA* and *lpfA* genes, they were previously identified in all *Salmonella* isolates in Brazil and Chile (Rowlands et al., 2014; Aravena et al., 2019). Both encode fimbriae-related proteins that carry essential adhesins that are associated with cell attachment to abiotic surfaces and biofilm formation for later intestinal colonization and expression of virulence, which indicates potential risk after infection (Yoo et al., 2013).

The *sefA* gene also encodes a fimbria-related protein (SefA) that is associated with the adhesion process. It is restricted to group D serovars, Enteritidis, Dublin, Moscow, and Blegdon serotypes (Amini et al., 2010). Thus, the absence of this gene in *S*H was expected, but its presence was investigated due to the possibility of genetic recombination, which can occur when the *sefABCD* genes present G + C around 35.2%, suggesting horizontal transfer (Bäumler et al., 1997; Mendonça et al., 2020).

The *avrA* gene encodes effector proteins essential for infection and bacterial proliferation, escaping the host’s immune system. This mechanism is possible through the induction of cellular apoptosis that limits the inflammatory response to infection (Labriola et al., 2018). It is a highly conserved gene in *Salmonella* spp. of public health importance, including *S*H (Borsoi et al., 2009).

Despite the high potential for adhesion and the biofilm initialization process, it is possible that part of the strains (30%–6/20) produce poorly stable structures or with low bacterial load due to the absence of the *luxS* gene, as observed comparatively for the H18 strain (*-luxS*) and H06 (+*luxS*) (Table 4). The LuxS protein is linked to the production of autoinducers that accumulate in the extracellular environment and signal the quorum sensing mechanism that allows bacterial reorientation aiming at ensuring the organization, the bacterial population at the quantitative level, the stability and maturity of the sessile structure (Parveen and Cornell, 2011). Although we observed similar biomass for the two strains, the bacterial count was significantly higher (*p* < 0.05) for the H06 sessile strain at temperatures of 25 and 37°C due to a probable expression of the *luxS* gene.

### Genetic Similarity Between Strains

Each pulsotype gathered two or three strains, demonstrating that they belong to the same origin (or producer) whose genotype was disseminated to different industrial units. The exception was pulsotype IV, whose strains had all the common epidemiological and molecular characteristics. In this case, the local maintenance of the microorganism in different samples can be linked to cross contamination (Melo et al., 2020).

The profile of antimicrobial resistance was variable within all pulsotypes, since it is a phenotypic characteristic that depends directly on the different ways in which the bacteria modulate the protein expression (Etter et al., 2019). Bacterial gene expression is regulated in order to prevent unnecessary protein production and its phenotype can undergo rapid changes according to the conditions to which they are exposed (Bhardwaj et al., 2021).

We emphasize the distinct pattern presented by the H08 strain, isolated from a 46-days old animal (older), characterized as the most phylogenetically distinct strain that was not grouped with any resistome and presented the greatest phenotypic susceptibility (P1 - Table 1). The greater susceptibility can be explained by the older age of the sampled chicken, due to a possible decrease in bacteria resistance to antimicrobials during the rearing period, when the use of antimicrobials is not done or is reduced (Moreno et al., 2019), different from the profile observed in younger chickens such as the H01 strain.

### Phenotype of Biofilms by *S*H

The sessile structures of *S*H showed an effective increase in their intensity, according to the BFI, at higher temperatures (25 and 37°C). According to de Melo et al. (2021), the use of higher temperatures also favors the production of biofilms in *S.* Minnesota. The influence of temperature on the establishment of biofilms is described as a serovar- and strain-dependent characteristic. Although thermal stress at low temperatures is an important trigger for the production of biofilms (Stepanović et al., 2003; Ivana et al., 2015; Piras et al., 2015), the test at 4°C may have made bacterial multiplication infeasible due to its proximity to the minimum growth temperature (Matches and Liston, 1968). The inclusion of CJ promoted a significant reinforcement in bacterial biomass, increasing on average 0.2114 the BFI when compared to the value of the non-supplemented samples, except at 4°C (Table 4). This increase can represent the major problem of food contamination. The use of biofilms analysis model with CJ approaches the condition only found in the environment processing of carcasses and represents the major source of bacterial contamination in the processing surfaces. CJ contains a complex mixture of carbohydrates (0.06%) and protein (2.79%), giving an ideal medium for the proliferation and survival of bacteria and allows the formation of micro-layers on the surfaces that aid in bacterial fixation. The influence of the increase promoted by the CJ was also identified in the *Campylobacter jejuni, S.* Typhimurium, *S.* Enteritidis, and *S.* Minnesota (Cappitelli et al., 2014; Li et al., 2017; Melo et al., 2017; de Melo et al., 2021). Despite the increase of extracellular matrix, CJ did not increase the number of viable cells, which is a strain-dependent characteristic.

Scanning electron microscope showed that the two strains of *S*H produce similar biofilm structure at the different temperatures tested. At 25 and 37°C, the structure of the biofilm matrix was very similar, with a more compact and stable architecture, in addition to the presence of a regular coverage along the surface, consistent with the maintenance of several microcolonies. In contrast, at 4°C, only the production of a few extracellular matrix fibers was observed, indicating the initial stage of biofilm formation. The closed ultra-structure composed of channels indicates a homogeneous biofilm, typical of the mature sessile structure with the presence of interconnections that assist in the nutrient distribution inside the cellular aggregates, as well as in the drainage of metabolic waste (Donlan and Costerton, 2002).

In the biofilm inhibition test, the three tested chemical agents, widely used in the industrial routine, reduced the number of sessile cells. Still, none of them was capable of eliminating all the microorganism (Figure 3). Tolerance to different sanitizers suggests the development of intrinsic or extrinsic adaptive mechanisms allowing the survival of *S*H, arising from the inappropriate use in the industrial routine due sublethal exposure to these biocides that can lead to bacterial adaptation. Also, it can positively influence biofilm production, promoting bacterial survival even in harsh environments, since the bacterial adaptive response mechanisms to stress are activated in these conditions (Pereira, 2014; Capita et al., 2017).

The efficiency of a chemical sanitizing agent is proven by a 3.0 log reduction in the count of bacterial cells. Thus, sodium hypochlorite 1% was the only product that did not reach this score since it promoted an average of 2.4 log reduction. The low effectiveness detected for this agent can also be associated with molecular factors such as the presence of *RpoS* and *Dps* genes, which were identified in both strains (H06 and H18) by WGS in our study. These oxidative stress-related genes are highly expressed in sodium hypochlorite-resistant *S.* enterica strains at a concentration of 200 ppm. In parallel, the properties of this sanitizer can be altered according to the pH variation and with the presence of organic matter that alternates the availability of hypochlorous acid, reducing its efficiency (Allan Pfuntner, 2011; Ritter et al., 2012).

Although the use of chemical compounds provides benefits to disinfection, these agents have the limitation of not destroying the residual structures of the biofilm matrix, which can facilitate bacterial resurgence or even the maintenance of these structures on the surfaces. Thus, special efforts are required for the complete removal of *S*H biofilms adapted to biocides. Probably, hygiene plans that combine cleaning measures focused on eliminating the extra polymeric matrix combined with the use of different agents, and the periodic rotation of sanitizers, respecting the periods between sanitizations, will be efficient in the growth control of these microorganisms (Ohsumi et al., 2015; Cardoso, 2019).

### Prediction in *S*H

Antimicrobial resistance and biofilm formation have been linked to the virulence and persistence of bacterial isolates (Alcántar-Curiel et al., 2018). The protein-protein interaction (PPI) results show that 41 of the selected 44 proteins form an association network (Figure 5). Three main clusters can be visualized in the network (Figure 5 – 1, 2, and 3).

Cluster 1 included the complete set of genes involved in the production of Curli proteins. The Curli protein is a type of extracellular fiber produced by some bacteria that serves to promote cell adhesion and invasion, biofilm formation, and environmental persistence. Curli assembly involves the six proteins encoded by the curli specific genes (*csg*) A, B, D, E, F, and G (Green et al., 2016).

Cluster 2 combines proteins related to fimbrial proteins and fimbrium biogenesis. One of the subnetworks inside cluster 2 is associated to the Lpf complex (A, B, C, D, and E). Proteins FimA, FimC, FimF, FimH, and FimI forms the other subnetwork. Both systems are directly related to the adhesion mechanism present in *S. enterica.* They are also involved in both pathogenesis and environmental persistence through the formation of biofilms. The Fim and Lpf complexes belong to the same group of fimbriae (type I) and therefore were grouped in the same cluster (Wagner and Hensel, 2011).

Cluster 3 showed the involvement of two key metabolic pathways (flagellar assembly and bacterial chemotaxis), shown in Figure 5 part 3. This interaction network encompasses the pathways associated with flagellar biogenesis.

Bacterial flagellum is an incredibly complex molecular machine with a variety of functions in the pathogenesis, including reaching the target host cell, colonization or invasion, maintenance at the site of infection and post-infection dispersal. Even though flagellum has generally been associated only with motility, it can involve other biological functions. In particular, flagella have also been reported to function as adhesins, having critical action in bacterial adhesion and virulence (Haiko and Westerlund-Wikström, 2013). The flagellar engine also responds to the chemotaxis system (represented in our study by 6 proteins in the network). This system allows the pathogen to escape from hostile environments through movement redirection. In addition to the motility function, the flagellum can also assist in adhesion to surfaces, biofilm formation, secretion of effector molecules, tissue penetration, or in the activation of phagocytosis to enter the eukaryotic cells (Chaban et al., 2015).

The interaction between clusters 1 and 3 identified the involvement of the flagellar apparatus combined with two-component systems (TCS). TCS is an adaptive mechanism to coordinate and alter bacterial gene expression. It is involved in motility, virulence, nutrient acquisition, biofilm production, and antimicrobial responses (Shetty et al., 2019; Tierney and Rather, 2019; Murret-Labarthe et al., 2020).

These results demonstrate that all predicted protein-protein networks have a direct connection between virulence factors and biofilm formation.

## Conclusion

The molecular and phenotypic diversity of *S*H isolated from chicken-associated products is still rarely explored in Brazil, and the present study represents a step forward with its characterization. Our results provide information related to distribution of virulent profiles and multidrug-resistant *S*H.

In addition to the presence of mobile genetic elements that encoded phenotypic resistance to penicillin, ceftriaxone, cefoxitin, and meropenem, the alarming resistance to non-lactam drugs is also observed, which include tetracycline, sulfamethoxazole, and colistin. These data are worrisome due to potential transmission to humans at the end of the food chain.

In parallel, the genotypic plasticity identified in the similarity analysis combined with the ability to form biofilms under different temperatures and especially in conditions that mimic the industrial environment, as well as the tolerance to biocidal agents in this lifestyle, can contribute to the persistence of strains along the food chain.

An evidence of presence of two metabolic pathways linked to the processes of virulence and biofilm formation highlights the need for special attention to this serotype in broiler production, with the inclusion of strict protocols of monitoring.

## Data Availability Statement

Raw sequence reads for strains H6 and H18 are available in DDBJ/ENA/GenBank under the accession numbers PRJNA707436 and PRJNA707472, respectively.

## Author Contributions

RM, NG, MG-T, and RP wrote the manuscript. RM, BF, VA, and DR designed the study. NG, RM, MG-T, PP, and GM experimental work about biofilms, antimicrobial resistance and molecular analyses. RP, RM, and BB conducted *in silico* analyses and interpreted the results. MG-T, BF, VA, and PP critically reviewed and revised the manuscript. RM, RP, and DR supervised the study. All authors approved this manuscript for publication.

## Conflict of Interest

The authors declare that the research was conducted in the absence of any commercial or financial relationships that could be construed as a potential conflict of interest.

## Footnotes

1 https://cge.cbs.dtu.dk/services/ResFinder/

2 https://www.genome.jp/kegg/pathway.html

3 https://www.ncbi.nlm.nih.gov/pathogens/
