# Supplementary material for: Molecular Characterization and Survive Abilities of Salmonella Heidelberg Strains of Poultry Origin in Brazil
Source: Front Microbiol. 2021 Jun 18;12:674147. doi: 10.3389/fmicb.2021.674147 (PMC8253257; doi:10.3389/fmicb.2021.674147)
Supplement: Supplementary file 1 [file Table_1.DOCX]

# Supplementary Material

Supplementary Table 1: List of virulence genes predicted in the genomes of Salmonella Heidelberg H6 and H18

| Virulence genes | Product | Strain |
| --- | --- | --- |
| *avrA* | type III secretion system effector AvrA acetyltransferease | both |
| *mig-14* | antimicrobial peptide resistance protein Mig-14 | both |
| *pipB2* | type III secretion system effector PipB3 | both |
| *iroN* | salmochelin receptor IroN | both |
| *iroC* | ATP binding cassette transporter | both |
| *iroB* | glucosyltransferase IroB | both |
| *sspH2* | type III secretion system effector SspH2 E3 ubiquitin ligase | both |
| *sseK2* | type III secretion system effector SseK2 | both |
| *sopA* | type III secretion system effector SopA E3 ubiquitin ligase | both |
| *fliP* | flagellar biosynthetic protein FliP | both |
| *fliM* | flagellar motor switch protein FliM | both |
| *fliG* | flagellar motor switch protein G | both |
| *fliA* | flagellar biosynthesis sigma factor | both |
| *flhC* | flagellar biosynthesis transcription activator FlhC | both |
| *cheW* | purine-binding chemotaxis protein CheW | both |
| *cheY* | chemotaxis regulatory protein CheY | both |
| *sopE2* | type III secretion system effector SopE2 guanine nucleotide exchange factor | both |
| *steC* | type III secretion system effector SteC | both |
| *sseJ* | type III secretion system effector SseJ glycerophospholipid:cholesterol acyltransferase | both |
| *steB* | type III secretion system effector SteB | both |
| *sifB* | type III secretion system effector SifB | both |
| *steA* | type III secretion system effector SteA | both |
| *ssaU* | type III secretion system export apparatus switch protein SsaU | both |
| *ssaT* | type III secretion system minor export apparatus protein SsaT | both |
| *ssaS* | type III secretion system minor export apparatus protein SsaS | both |
| *ssaR* | type III secretion system minor export apparatus protein SsaR | both |
| *ssaQ* | type III secretion system C ring protein SsaQ | both |
| *ssaP* | type III secretion system needle length regulator SsaP | both |
| *ssaO* | type III secretion system stalk protein SsaO | both |
| *ssaN* | type III secretion system ATPase SsaN | both |
| *ssaV* | type III secretion system major export apparatus protein ssaV | both |
| *ssaM* | type III secretion system protein SsaM | both |
| *ssaL* | type III secretion system gatekeeper SsaL | both |
| *ssaK* | type III secretion system stator SsaK | both |
| *ssaJ* | type III secretion system inner MS ring protein SsaJ | both |
| *ssaI* | type III secretion system inner rod protein SsaI | both |
| *ssaH* | type III secretion system protein SsaH | both |
| *ssaG* | type III secretion system needle filament protein SsaG | both |
| *sseG* | type III secretion system effector SseG | both |
| *sseF* | type III secretion system effector SseF | both |
| *sscB* | chaperone for sseF | both |
| *sseE* | type III secretion system effector SseE | both |
| *sseD* | type III secretion system hydrophilic translocator pore protein SseD | both |
| *sseC* | type III secretion system hydrophilic translocator pore protein SseC | both |
| *sscA* | chaperone for sseC | both |
| *sseB* | type III secretion system effector SseB | both |
| *sseA* | chaperone for sseB and sseD | both |
| *ssaE* | chaperone for sseB | both |
| *ssaD* | type III secretion system outer MS ring protein SsaD | both |
| *ssaC* | type III secretion system secretin SsaC | both |
| *spiC/ssaB* | type III secretion system protein SsaB | both |
| *slrP* | type III secretion system effector SlrP E3 ubiquitin ligase | both |
| *entA* | 23-dihydro-23-dihydroxybenzoate dehydrogenase | both |
| *entB* | isochorismatase | both |
| *entE* | 23-dihydroxybenzoate-AMP ligase component of enterobactin synthase multienzyme complex | both |
| *entC* | isochorismate synthase 1 | both |
| *fepB* | ferrienterobactin ABC transporter periplasmic binding protein | both |
| *entS* | enterobactin exporter iron-regulated | both |
| *fepD* | ferrienterobactin ABC transporter permease | both |
| *fepG* | iron-enterobactin ABC transporter permease | both |
| *fepC* | ferrienterobactin ABC transporter ATPase | both |
| *fepA* | ferrienterobactin outer membrane transporter | both |
| *gtrB* | bactoprenol glucosyl transferase | both |
| *fimF* | type I fimbriae adaptor protein FimF | both |
| *fimH* | type I fimbriae minor fimbrial subunit FimH adhesin | both |
| *fimD* | usher protein FimD | both |
| *fimC* | chaperone protein FimC | both |
| *fimI* | fimbrial protein internal segment | both |
| *sopD* | type III secretion system effector SopD | both |
| *invH* | type III secretion system pilotin invG | both |
| *invF* | type III secretion system regulatory protein InvF | both |
| *invG* | type III secretion system secretin invG | both |
| *invE* | type III secretion system gatekeeper invE | both |
| *invA* | type III secretion system major export apparatus protein InvA | both |
| *invB* | type III secretion system protein InvB | both |
| *invC* | type III secretion system ATPase InvC | both |
| *invI* | type III secretion system stalk protein InvI | both |
| *invJ* | type III secretion system needle length regulator InvJ | both |
| *spaO* | type III secretion system C ring protein SpaO | both |
| *spaP* | type III secretion system minor export apparatus protein SpaP | both |
| *spaQ* | type III secretion system minor export apparatus protein SpaQ | both |
| *spaR* | type III secretion system minor export apparatus protein SpaR | both |
| *spaS* | type III secretion system export apparatus switch protein SpaS | both |
| *sicA* | chaparone for SipC and SipB | both |
| *sipB/sspB* | type III secretion system hydrophilic translocator pore protein SipB | both |
| *sipC/sspC* | type III secretion system hydrophilic translocator pore protein SipC | both |
| *sipD* | type III secretion system hydrophilic translocator needle tip protein SipD | both |
| *sipA/sspA* | type III secretion system effector SipA actin polymerizing activity | both |
| *sicP* | chaparone for SptP | both |
| *lpfE* | long polar fimbrial minor subunit LpfE adhesin | both |
| *lpfD* | long polar fimbrial protein LpfD | both |
| *lpfC* | long polar fimbrial usher protein LpfC | both |
| *lpfB* | long polar fimbrial chaperone protein LpfB | both |
| *lpfA* | long polar fimbria protein LpfA | both |
| *misL* | putative autotransporter | both |
| *mgtB* | Mg2+ transport protein | both |
| *mgtC* | Mg2+ transport protein | both |
| *sseL* | type III secretion system effector SseL deubiquitinase | both |
| *sifA* | type III secretion system effector SifA N-terminal SKIP-binding domain C-terminal guanine nucleotide exchange factor activity | both |
| *flgH* | flagellar L-ring protein precursor FlgH | both |
| *flgG* | flagellar basal-body rod protein FlgG | both |
| *csgC* | curli assembly protein CsgC | both |
| *csgA* | curlin major subunit CsgA | both |
| *csgB* | minor curlin subunit precursor curli nucleator protein CsgB | both |
| *csgD* | DNA-binding transcriptional regulator CsgD | both |
| *csgE* | curli production assembly/transport protein CsgE | both |
| *csgF* | curli production assembly/transport protein CsgF | both |
| *csgG* | curli production assembly/transport protein CsgG | both |
| *sopB/sigD* | type III secretion system effector SopB phosphoinositide phosphatase | both |
| *pipB* | type III secretion system effector PipB | both |
| *ompA* | outer membrane protein A | both |
| *sodCI* | Gifsy-2 prophage: superoxide dismutase precursor (Cu-Zn | both |
| *grvA* | gifsy-2 related virulence gene | both |
| *sopD2* | type III secretion system effector SopD2 | both |
| *sinH* | intimin-like protein | both |
| *ratB* | putative outer membrane protein | both |
| *shdA* | AIDA autotransporter-like protein | both |
| *fyuA* | pesticin/yersiniabactin receptor protein | both |
| *ybtE* | yersiniabactin siderophore biosynthetic protein | both |
| *ybtT* | yersiniabactin biosynthetic protein YbtT | both |
| *ybtU* | yersiniabactin biosynthetic protein YbtU | both |
| *irp1* | yersiniabactin biosynthetic protein Irp1 | both |
| *irp2* | yersiniabactin biosynthetic protein Irp2 | both |
| *ybtA* | transcriptional regulator YbtA | both |
| *ybtP* | lipoprotein inner membrane ABC-transporter | both |
| *ybtQ* | inner membrane ABC-transporter YbtQ | both |
| *ybtX* | putative signal transducer | both |
| *sptP* | type III secretion system effector SptP tyrosine phosphatase and GTPase-activating protein | H18 only |
| *prgH* | type III secretion system outer MS ring protein PrgH | H18 only |
| *prgI* | type III secretion system needle filament protein PrgI | H18 only |
| *prgJ* | type III secretion system inner rod protein PrgJ | H18 only |
| *prgK* | type III secretion system inner MS ring protein PrgK | H18 only |
| *orgA* | type III secretion system accessory cytosolic protein OrgA | H18 only |
| *orgB* | type III secretion system stator OrgB | H18 only |
| *orgC* | type III secretion system effector OrgC | H18 only |

**Supplementary Table 2:** List of selected proteins for protein-protein interaction prediction

| **Gene** | **Product** |
| --- | --- |
| *cheA* | Chemotaxis protein CheA |
| *cheW* | Chemotaxis protein CheW |
| *cheY* | Two-component system response regulator; Chemotaxis regulator that, when phosphorylated, interacts with the flagellar motor causing the flagella to spin clockwise which causes the cell to tumble |
| *cheZ* | Protein phosphatase CheZ; Plays an important role in bacterial chemotaxis signal transduction pathway by accelerating the dephosphorylation of phosphorylated CheY (CheY-P) |
| *csgA* | Major curlin subunit CsgA |
| *csgB* | Curlin minor subunit CsgB; CsgB; functions as a nucleator in the assembly of curli (coiled surface structures) on the cell surface |
| *csgD* | Transcriptional regulator CsgD; Activates the csgBA and csgDEFG operons involved in biofilm formation |
| *csgE* | Curli production assembly/transport component CsgE; Chaperone-like protein that participates in the polymerization of curlin (CsgA) subunits into curli (extracellular fibers from Escherichia and Salmonella spp. that are involved in the colonization of inert surfaces and biofilm formation); part of the curli secretion and assembly protein complex |
| *csgF* | Curli production assembly/transport component CsgF; Nucleator protein that participates in the polymerization of curlin (CsgA) subunits into curli (extracellular fibers from Escherichia and Salmonella spp. that are involved in the colonization of inert surfaces and biofilm formation); part of the curli secretion and assembly protein complex |
| *csgG* | Curli production assembly/transport component CsgG; Involved in the stability of the curlin proteins during assembly; involved in the secretion of the major curlin subunit CsgA across the outer membrane |
| *fimC* | Fimbrial chaperone protein FimC; Involved in type 1 fimbriae biosynthesis, interacts with FimH |
| *fimD* | Outer membrane usher protein |
| *fimF* | Fimbrial-like protein FimF |
| *fimH* | FimH protein; Involved in the regulation of fimbriae length and mediates adhesion of type 1 fimbriae |
| *fimI* | Type 1 fimbrial protein subunit FimI |
| *flgB* | Flagellar basal body rod protein FlgB; Structural component of flagellum, the bacterial motility apparatus. Part of the rod structure of flagellar basal body |
| *flgC* | Flagellar basal-body rod protein FlgC; With FlgF and B makes up the proximal portion of the flagellar basal body rod |
| *flgD* | Basal-body rod modification protein FlgD; Required for flagellar hook formation. May act as a scaffolding protein |
| *flgE* | The hook connects flagellar basal body to the flagellar filament |
| *flgF* | Flagellar basal body protein; FlgF, with FlgB and C, makes up the proximal portion of the flagellar basal body rod |
| *flgG* | Flagellar basal-body rod protein FlgG; Makes up the distal portion of the flagellar basal body rod |
| *flgH* | Flagellar L-ring protein; Assembles around the rod to form the L-ring and probably protects the motor/basal body from shearing forces during rotation |
| *flgI* | Flagellar P-ring protein; Assembles around the rod to form the L-ring and probably protects the motor/basal body from shearing forces during rotation |
| *flgJ* | Flagellar assembly peptidoglycan hydrolase FlgJ; Flagellum-specific muramidase which hydrolyzes the peptidoglycan layer to assemble the rod structure in the periplasmic space |
| *flgK* | Flagellar hook-associated protein 1; With FlgL acts as a hook filament junction protein to join the flagellar filament to the hook |
| *flhC* | Flagellar transcriptional regulator FlhC; Functions in complex with FlhD as a master transcriptional regulator that regulates transcription of several flagellar and non-flagellar operons by binding to their promoter region. Activates expression of class 2 flagellar genes, including fliA, which is a flagellum-specific sigma factor that turns on the class 3 genes. Also regulates genes whose products function in a variety of physiological pathways; Belongs to the FlhC family |
| *flhD* | Flagellar transcriptional regulator FlhD; Functions in complex with FlhC as a master transcriptional regulator that regulates transcription of several flagellar and non-flagellar operons by binding to their promoter region. Activates expression of class 2 flagellar genes, including fliA, which is a flagellum-specific sigma factor that turns on the class 3 genes. Also regulates genes whose products function in a variety of physiological pathways; Belongs to the FlhD family |
| *fliA* | RNA polymerase sigma factor FliA; Sigma factors are initiation factors that promote the attachment of RNA polymerase to specific initiation sites and are then released. This sigma factor controls the expression of flagella-related genes |
| *fliF* | Flagellar M-ring protein; The M ring may be actively involved in energy transduction |
| *fliG* | One of three proteins involved in switching the direction of the flagellar rotation |
| *fliH* | Flagellar assembly protein FliH; Binds to and inhibits the function of flagella specific ATPase FliI |
| *fliK* | Flagellar hook-length control protein |
| *fliL* | Flagellar protein FliL; Controls the rotational direction of flagella during chemotaxis |
| *fliM* | Flagellar motor switch protein FliM; FliM is one of three proteins (FliG, FliN, FliM) that forms the rotor-mounted switch complex (C ring), located at the base of the basal body. This complex interacts with the CheY and CheZ chemotaxis proteins, in addition to contacting components of the motor that determine the direction of flagellar rotation |
| *fliP* | Flagellar biosynthetic protein FliP; Plays a role in the flagellum-specific transport system |
| *lpfA* | Long polar fimbrial protein LpfA |
| *lpfB* | Long polar fimbrial chaperone LpfB; Binds with fimbrial subunit LpfA |
| *lpfC* | Outer membrane usher protein LpfC |
| *lpfD* | Long polar fimbrial protein LpfD |
| *lpfE* | Long polar fimbrial protein LpfE |
| *mig-14* | Induced within macrophages; necessary for the resistance to antimicrobials |
| *nleB* | Non-LEE encoded effector protein NleB |
| *ompA* | Outer membrane protein A; OmpA is believed to be a porin, involved in diffusion of nonspecific small solutes across the outer membrane. It is the most abundant integral protein of the outer membrane of E. coli, and it is known to play a role as a phage receptor, a mediator of F-factor dependent conjugation, and in maintaining the structural shape of the outer membrane; 3a; II*; G; d |
| *spiC* | SPI-2 type III secretion system protein SpiC; Involved in macrophage infection; inhibits phagosome-lysosome fusion and cellular trafficking |

| **Supplementary Table 3.** Product description of all resistance genes found in the strains 6H and 18H   \|  \| Resistance genes \| Product \| \| --- \| --- \| --- \| \| 1 \| *cpxA* \| CpxA is a membrane-localized sensor kinase that is activated by envelope stress. It starts a kinase cascade that activates CpxR which promotes efflux complex expression. \| \| 2 \| *ANT(3'')-IIa* \| ANT(3'')-IIa is a aminoglycoside nucleotidyltransferase identified in Acinetobacter spp. via horizontal gene transfer mechanisms. \| \| 3 \| *AAC(3)-VIa* \| AAC(3)-VIa is a plasmid-encoded aminoglycoside acetyltransferase in E. cloacae S. enterica and E. coli \| \| 4 \| *sul1* \| Sul1 is a sulfonamide resistant dihydropteroate synthase of Gram-negative bacteria. It is linked to other resistance genes of class 1 integrons. \| \| 5 \| *CTX-M-2* \| CTX-M-2 is a beta-lactamase found in the Enterobacteriaceae family \| \| 6 \| *emrB* \| emrB is a translocase in the emrB -TolC efflux protein in E. coli. It recognizes substrates including carbonyl cyanide m-chlorophenylhydrazone (CCCP) nalidixic acid and thioloactomycin. \| \| 7 \| *emrA* \| EmrA is a membrane fusion protein providing an efflux pathway with EmrB and TolC between the inner and outer membranes of E. coli a Gram-negative bacterium. \| \| 8 \| *emrR* \| EmrR is a negative regulator for the EmrAB-TolC multidrug efflux pump in E. coli. Mutations lead to EmrAB-TolC overexpression. \| \| 9 \| *acrF* \| AcrF is a inner membrane transporter similar to AcrB. \| \| 10 \| *acrE* \| AcrE is a membrane fusion protein similar to AcrA. \| \| 11 \| *baeR* \| BaeR is a response regulator that promotes the expression of MdtABC and AcrD efflux complexes. \| \| 12 \| *baeS* \| BaeS is a sensor kinase in the BaeSR regulatory system. While it phosphorylates BaeR to increase its activity BaeS is not necessary for overexpressed BaeR to confer resistance. \| \| 13 \| *mdtC* \| MdtC is a transporter that forms a heteromultimer complex with MdtB to form a multidrug transporter. MdtBC is part of the MdtABC-TolC efflux complex. In the absence of MdtB MdtC can form a homomultimer complex that results in a functioning efflux complex with a narrower drug specificity. mdtC corresponds to 3 loci in Pseudomonas aeruginosa PAO1 (gene name: muxC/muxB) and 3 loci in Pseudomonas aeruginosa LESB58. \| \| 14 \| *mdtB* \| MdtB is a transporter that forms a heteromultimer complex with MdtC to form a multidrug transporter. MdtBC is part of the MdtABC-TolC efflux complex. \| \| 15 \| *mdtA* \| MdtA is the membrane fusion protein of the multidrug efflux complex mdtABC. \| \| 16 \| *ugd* \| PmrE is required for the synthesis and transfer of 4-amino-4-deoxy-L-arabinose (Ara4N) to Lipid A which allows gram-negative bacteria to resist the antimicrobial activity of cationic antimicrobial peptides and antibiotics such as polymyxin \| \| 17 \| *mdtK* \| A multidrug and toxic compound extrusions (MATE) transporter conferring resistance to norfloxacin doxorubicin and acriflavine. \| \| 18 \| *sdiA* \| SdiA is a cell division regulator that is also a positive regulator of AcrAB only when it's expressed from a plasmid. When the sdiA gene is on the chromosome it has no effect on expression of acrAB \| \| 19 \| *H-NS* \| H-NS is a histone-like protein involved in global gene regulation in Gram-negative bacteria. It is a repressor of the membrane fusion protein genes acrE mdtE and emrK as well as nearby genes of many RND-type multidrug exporters. \| \| 20 \| *AAC(6')-Iy* \| AAC(6')-Iy is a chromosomal-encoded aminoglycoside acetyltransferase in S. enteritidis and S. enterica. Regulatory mutation required to increase expression of this chromosomally-encoded gene for resistance. In the specific system aminoglycoside resistance was due to a transcriptional fusion secondary to a chromosomal deletion in which the downstream aac(6')-Iy gene was placed under the control of the upstream nmpC promoter. \| \| 21 \| *marA* \| In the presence of antibiotic stress E. coli overexpresses the global activator protein MarA which besides inducing MDR efflux pump AcrAB also down- regulates synthesis of the porin OmpF. \| \| 22 \| *KpnE* \| KpnE subunit of KpnEF resembles EbrAB from E. coli. Mutation in KpnEF resulted in increased susceptibility to cefepime ceftriaxon colistin erythromycin rifampin tetracycline and streptomycin as well as enhanced sensitivity toward sodium dodecyl sulfate deoxycholate dyes benzalkonium chloride chlorhexidine and triclosan \| \| 23 \| *OmpK37* \| Klebsiella pneumoniae outer membrane porin protein. Is preferentially detected in porin-deficient strains. Functional characterization of this new porin revealed a narrower pore than those of porins OmpK35 and OmpK36 which did not allow penetration by certain beta-lactams. Also when a resistant strain expresses porin OmpK37 is less susceptible to cefotaxime and cefoxitin than when it is expressing either OmpK36 or OmpK35. \| \| 24 \| *mdfA* \| Multidrug efflux pump in E. coli. This multidrug efflux system was originally identified as the Cmr/CmlA chloramphenicol exporter. \| \| 25 \| *kdpE* \| kdpE is a transcriptional activator that is part of the two-component system KdpD/KdpE that is studied for its regulatory role in potassium transport and has been identified as an adaptive regulator involved in the virulence and intracellular survival of pathogenic bacteria. kdpE regulates a range of virulence loci through direct promoter binding. \| \| 26 \| *ramA* \| RamA (resistance antibiotic multiple) is a positive regulator of AcrAB-TolC and leads to high level multidrug resistance in Klebsiella pneumoniae Salmonella enterica and Enterobacter aerugenes increasing the expression of both the mar operon as well as AcrAB. RamA also decreases OmpF expression. \| \| 27 \| *acrA* \| AcrA is a subunit of the AcrAB-TolC multidrug efflux system that in E. coli. \| \| 28 \| *acrB* \| Protein subunit of AcrA-AcrB-TolC multidrug efflux complex. AcrB functions as a herterotrimer which forms the inner membrane component and is primarily responsible for substrate recognition and energy transduction by acting as a drug/proton antiporter. \| \| 29 \| *ampH* \| AmpH is a class C ampC-like beta-lactamase and penicillin-binding protein identified in Escherichia coli. \| \| 30 \| *golS* \| GolS is a regulator activated by the presence of golD and promotes the expression of the MdsABC efflux pump. \| \| 31 \| *mdsA* \| MdsA is the membrane fusion protein of the multidrug and metal efflux complex MdsABC. \| \| 32 \| *mdsB* \| MdsB is the inner membrane transporter of the multidrug and metal efflux complex MdsABC. mdsB corresponds to 1 locus in Pseudomonas aeruginosa PAO1 (gene name: mexQ) and 2 loci in Pseudomonas aeruginosa LESB58. \| \| 33 \| *mdsC* \| MdsC is the outer membrane channel of the multidrug and metal efflux complex MdsABC. \| \| 34 \| *bacA* \| The bacA gene product (BacA) recycles undecaprenyl pyrophosphate during cell wall biosynthesis which confers resistance to bacitracin. \| \| 35 \| *tolC* \| TolC is a protein subunit of many multidrug efflux complexes in Gram negative bacteria. It is an outer membrane efflux protein and is constitutively open. Regulation of efflux activity is often at its periplasmic entrance by other components of the efflux complex. \| \| 36 \| *CRP* \| CRP is a global regulator that represses MdtEF multidrug efflux pump expression. \| \| 37 \| *yojI* \| YojI mediates resistance to the peptide antibiotic microcin J25 when it is expressed from a multicopy vector. YojI is capable of pumping out microcin molecules. The outer membrane protein TolC in addition to YojI is required for export of microcin J25 out of the cell. Microcin J25 is thus the first known substrate for YojI. \| \| 38 \| *pmrF* \| PmrF is required for the synthesis and transfer of 4-amino-4-deoxy-L-arabinose (Ara4N) to Lipid A which allows gram-negative bacteria to resist the antimicrobial activity of cationic antimicrobial peptides and antibiotics such as polymyxin. pmrF corresponds to 1 locus in Pseudomonas aeruginosa PAO1 and 1 locus in Pseudomonas aeruginosa LESB58. \| \| 39 \| *mdtH* \| Multidrug resistance protein MdtH \| \| 40 \| *mdtG* \| The MdtG protein also named YceE appears to be a member of the major facilitator superfamily of transporters and it has been reported when overexpressed to increase fosfomycin and deoxycholate resistances. mdtG is a member of the marA-soxS-rob regulon. \| \| 41 \| *msbA* \| MsbA is a multidrug resistance transporter homolog from E. coli and belongs to a superfamily of transporters that contain an adenosine triphosphate (ATP) binding cassette (ABC) which is also called a nucleotide-binding domain (NBD). MsbA is a member of the MDR-ABC transporter group by sequence homology. MsbA transports lipid A a major component of the bacterial outer cell membrane and is the only bacterial ABC transporter that is essential for cell viability. \| \| 42 \| *eptA* \| PmrC mediates the modification of Lipid A by the addition of 4-amino-4-deoxy-L-arabinose (L-Ara4N) and phosphoethanolamine resulting in a less negative cell membrane and decreased binding of polymyxin B. \| \| 43 \| *FosA7* \| fosA7 is an enzyme that confers resistance to fosfomycin in Escherichia coli by breaking the epoxide ring of the molecule. \| \| 44 \| *mdtM* \| Multidrug resistance protein MdtM \| \| 45 \| *acrD* \| AcrD is an aminoglycoside efflux pump expressed in E. coli. Its expression can be induced by indole and is regulated by baeRS and cpxAR. \| \| 46 \| *tet(A)* \| TetA is a tetracycline efflux pump found in many species of Gram-negative bacteria. \| \| 47 \| *sul2* \| Sul2 is a sulfonamide resistant dihydropteroate synthase of Gram-negative bacteria usually found on small plasmids. \| \| 48 \| *CMY-59* \| CMY-59 is a beta-lactamase found in Shigella spp. \| |  |  |
| --- | --- | --- | --- | --- | --- | --- | --- | --- | --- | --- | --- | --- | --- | --- | --- | --- | --- | --- | --- | --- | --- | --- | --- | --- | --- | --- | --- | --- | --- | --- | --- | --- | --- | --- | --- | --- | --- | --- | --- | --- | --- | --- | --- | --- | --- | --- | --- | --- | --- | --- | --- | --- | --- | --- | --- | --- | --- | --- | --- | --- | --- | --- | --- | --- | --- | --- | --- | --- | --- | --- | --- | --- | --- | --- | --- | --- | --- | --- | --- | --- | --- | --- | --- | --- | --- | --- | --- | --- | --- | --- | --- | --- | --- | --- | --- | --- | --- | --- | --- | --- | --- | --- | --- | --- | --- | --- | --- | --- | --- | --- | --- | --- | --- | --- | --- | --- | --- | --- | --- | --- | --- | --- | --- | --- | --- | --- | --- | --- | --- | --- | --- | --- | --- | --- | --- | --- | --- | --- | --- | --- | --- | --- | --- | --- | --- | --- | --- | --- | --- |
